# Toward Understanding Body Image Importance: Individual Differences in a Canadian Sample of Undergraduate Students

**DOI:** 10.1080/10640266.2013.761083

**Published:** 2013-02-19

**Authors:** Alexander B. Siegling, Mary E. Delaney

**Affiliations:** Research Department of Clinical, Educational, and Health Psychology, University College London, London, UK; Department of Psychology, Mount Saint Vincent University, Halifax, Nova Scotia, Canada

## Abstract

This study examined the relationships between body image importance (BII) andperfectionism and body satisfaction in a Canadian sample of undergraduate students. Specifically, perfectionism was conceptualized as a common cause of BII and body satisfaction. Furthermore, gender-schematic processing was examined as a moderator of sex differences in BII, which have been inconsistently found. As hypothesized, there was no significant partial correlation between BII and body satisfaction, controlling for perfectionism. Also, a significant Sex × Gender Schematicity interaction indicated that gender schematicity moderates sex differences in BII. Implications for understanding individual differences in, and elevated levels of BII are discussed.

Theoretical perspectives depict *overvalued ideas about shape and weight* as the core clinical feature of eating disorders (Cooper & Fairburn, 1993; Fairburn, Cooper, & Shafran, 2003), with individuals judging “their self-worth or value almost exclusively in terms of their shape and weight” (Fairburn & Cooper, 1989, p. 277). In addition to constituting a key diagnostic criterion for both anorexia and bulimia nervosa (American Psychiatric Association, 2000), research has shown that shape and weight overvaluation characterizes people with binge eating disorder (e.g., Masheb & Grilo, 2003; Wilfley, Schwartz, Spurrell, & Fairburn, 2000). However, this factor has been frequently ignored or confounded with body dissatisfaction (Cooper & Fairburn, 1993), despite research showing that it better differentiates between individuals with and without eating disorders (Goldfein, Walsh, & Midlarsky, 2000) and better predicts eating attitudes and behaviors than body dissatisfaction (Cash, Melnyk, & Hrabosky, 2004).

## Body Image Importance, Dissatisfaction, and Perfectionism

The construct *body image importance* (BII) has been used as a proxy for shape and weight overvaluation in research, particularly for non-clinical samples. BII has fewer evaluative connotations and is measured in relation to other aspects of the self (Rieder & Ruderman, 2001). An extensive line of research has demonstrated the role of BII in body change strategies and behaviors in non-clinical samples of Australian adolescents, indicating that mostly girls and boys who are high in BII engage in excessive body change behaviors (e.g., McCabe & Ricciardelli, 2003, 2006). Similar effects have been demonstrated cross-culturally (e.g., Muris, Meesters, van de Blom, & Mayer, 2005) and for undergraduate women (Rieder & Ruderman, 2001). The conceptualization of weight and shape overvaluation as a general, continuous construct (i.e., BII) facilitates the investigation of individual differences across populations. This knowledge will enrich the understanding of elevated levels of BII, which are conducive to maladaptive body change behaviors, and ultimately have implications for treatment.

Even though BII is a distinct construct (e.g., Allen, Byrne, McLean, & Davis, 2008; Cash et al., 2004), it does correlate with body dissatisfaction (e.g., McCabe & Ricciardelli, 2003; Rieder & Ruderman, 2001). It remains unclear, however, what precisely the nature of this relationship is (Allen et al., 2008); Shape and weight overvaluation may lead to greater body dissatisfaction and/or people high in body dissatisfaction may become increasingly more preoccupied with their bodies. Alternatively, the shared variance of these two constructs may be accounted for by a common cause. In addition to focusing on the associations between cognate body image constructs, investigations into the relations of these constructs with underlying cognitive and personality factors are needed.

Fairburn et al. (2003) consider specifically the cognitive aspects of perfectionism to be a maintaining mechanism of disturbed eating behavior, as outlined in their transdiagnostic theory.^1^ Their definition of clinical perfectionism (“the over-evaluation of the striving for, and achievement of, personally demanding standards, despite adverse consequences,” p. 515) conceptually resembles the core psychopathology of eating disorders (i.e., shape and weight overvaluation), with both being characterized by dysfunctional systems of self-evaluation. Weight and shape overvaluation is also empirically associated with perfectionism (e.g., Wade & Bulik, 2007) and has been identified as a mediator of the relationship between perfectionism and disturbed eating habits (Wade & Lowes, 2002; Watson, Raykos, Street, Fursland, & Nathan, 2011). Furthermore, perfectionism was moderately associated with body dissatisfaction in a study of female undergraduate students (Welch, Miller, Ghaderi, & Vaillancourt, 2009). Thus, perfectionism may not only explain individual differences in BII and body dissatisfaction but also account for the association of these body image facets.

## BII and Gender

Whereas women tend to be more dissatisfied with their bodies then men (e.g., Davison & McCabe, 2005; Feingold & Mazzella, 1998), sex differences in BII have been inconsistently found. In McCabe, Ricciardelli, and colleagues’ research of Australian adolescents, girls self-reported significantly higher BII scores than boys in some studies (e.g., McCabe & Ricciardelli, 2001, 2009), whereas in other studies no sex differences appeared (e.g., Davison & McCabe, 2006; McCabe & Ricciardelli, 2005). In one study, adolescent boys even self-reported higher BII scores than same-aged girls (McCabe, Ricciardelli, & Holt, 2010). In an investigation of an adult sample (age range 18–86 years), no sex difference was found (Davison & McCabe, 2005). While variations in statistical power and research design may contribute to the inconsistent findings, variables that were not previously examined may moderate sex differences in BII. As these investigations were conducted within the same cultural context, non-cultural factors may act as moderators.

Considering popular notions of the body as being “gendered,” individual differences in the reliance on gender as a cognitive organizing structure, or schema, may affect sex differences in BII. The *gender schema* is a cognitive structure consisting of a network of sex-linked associations, which represent society's definitions for masculinity and femininity (Bem, 1981a). According to Bem's gender schema theory, people assimilate their self-concept and evaluate their own adequacy relative to their gender schema, leading to modifications in their preferences, attitudes, and behaviors. Yet, people diverge in the reliance on the gender schema in processing information. Thus, *gender-schematic* individuals are more likely than *gender-aschematic* individuals to spontaneously encode and organize the social world on the basis of gender, and evaluate themselves and the world around them accordingly (Bem, 1981a). As the body is a central gender-defining property, gender schematicity could moderate the ambiguous relationship between sex and BII. Specifically, gender-schematic individuals may be driven to conform to gender roles regarding body shape and thus experience high BII, regardless of sex. In contrast, BII may be more variable amongst gender-aschematic individuals, especially for men, who are less likely to be evaluated by society based on appearance.

## Present Study

The aim of the present study was to examine the role of two cognitive individual-difference factors (cognitive perfectionism and gender schematicity) in BII. In particular, this study examined a) whether perfectionism explains the association between BII and body satisfaction, and b) if gender schematicity uniquely moderates sex differences in BII (and not in the related variables of body satisfaction and perfectionism). Previous investigations surrounding the BII construct were mainly conducted on children and early adolescents, whereas research has shown that body-related concerns are only established by mid-adolescence (Stice & Shaw, 2002). The present sample comprised individuals beyond this developmental stage, specifically undergraduate students in whom BII may be more stable. Consistent with the forgoing discussion of the literature, the following hypotheses were tested:

*Hypothesis_1_ (H1)*: There will be a negative correlation between BII and body satisfaction; however, a partial correlation between BII and body satisfaction, controlling for perfectionism, will not reach significance. An assumption for this hypothesis is that perfectionism will correlate positively with BII and negatively with body satisfaction, consistent with previous findings.

*H_2a_:* Gender schematicity will moderate potential sex differences in BII, which are expected to be more pronounced amongst gender-aschematic participants (females indicating higher BII than males).

*H_2b_:* By contrast, there will be no interaction of sex and gender schematicity on body satisfaction.

## METHOD

### Participants and Procedure

A convenience sample of 141 undergraduate students with a mean age of 20.18 years (*SD* = 2.53, age range 16–28 years) was recruited from introductory psychology courses at a small eastern Canadian university. The majority of participants were female (78.7%), reflecting the institution's student sex ratio, and between 18 and 23 years of age (85.9%).^2^ Based on the World Health Organization's (2010) *BMI classification*, 5.0% of the participants were Underweight (<18.50), 66.2% in the Normal range (18.50–24.99), 22.3% Overweight (25.00–29.99), and 5.8% in the Obese class 1 range (30.00–34.99). Two participants did not disclose their height and weight. The ethnic composition of the overall sample was uniform, with 87.2% of the participants classifying themselves as Caucasian, 3.8% as African Canadian/American, 2.1% as Asian, and 4.2% representing other ethnic groups (2.8% did not reveal their background). Participants provided demographic information and completed self-report measures of the constructs examined in this study.

### Measures

#### BMI

Self-reported height (ft) and weight (lbs) were used to calculate participants’ BMIs, using the formula (lbs*4.88)/ft^2^. The main purpose of obtaining an approximation of their BMIs was to better describe the study sample.

#### BII and Body Satisfaction

Two 10-item scales of the Body Image and Body Change Questionnaire (Ricciardelli & McCabe, 2002) were used to measure BII and body satisfaction. Items are rated on 5-point Likert scales that are labeled at each point. The labels range from *extremely important/dissatisfied* to *not important at all/extremely satisfied* (e.g., “How important is what you weigh compared to other things in your life?”, “How satisfied are you with your muscle size?”). High levels of internal consistency have been reported for both scales (αs > .90; e.g., Davison & McCabe, 2005). In the present study, BII and body satisfaction had alpha coefficients of .87 and .84, respectively.

#### Perfectionism

The cognitive dimension of perfectionism was assessed with the Perfectionism Cognitions Inventory (PCI; Flett, Hewitt, Blankstein, & Gray, 1998), a unidimensional, 25-item measure of the automatic thoughts that characterize perfectionists. Respondents indicate how often various perfectionist thoughts occurred to them over the last week (e.g., “I should be doing more”) on 5-point scales. Responses range from 0 (*not at all*) to 4 (*all of the time*) and are labeled at each point. The PCI yields a high level of internal consistency (α = .95) and incremental validity over trait perfectionism (Flett, Hewitt, Whelan, & Martin, 2007). The internal consistency was similarly high in the present sample (α = .93).

#### Gender Schematicity

Although originally designed to assess masculinity and femininity, the Bem Sex Role Inventory (BSRI; Bem, 1974) is also a valid measure of gender schematicity (Bem, 1981b; Schmitt & Millard, 1988). In particular, gender-schematic individuals (i.e., those with discrepant masculinity and femininity scores) respond to the BSRI's masculine (e.g., aggressive, forceful) and feminine items (e.g., compassionate, affectionate) as a function of their gender-schematic processing (Bem, 1981b). Items are responded to on 7-point Likert scales, which range from 1 *(never or almost never true)* to 7 *(always or almost always true)* and are labeled at each point. Participants also rated themselves on the BSRI's 20 neutral items (e.g., friendly, conscientious) to maintain the psychometric integrity of the scores. Using Bem's (1974) *t* ratio-scoring procedure,^3^ participants with significantly different masculinity and femininity scale means were classified as gender-schematic (72.3%), whereas participants who had similar masculinity and femininity scores were classified as gender-aschematic (27.7%). Bem reported high levels of internal consistency for both scales (masculinity α = .86, femininity α = .80–.82), resembling those of the current sample (masculinity α = .85, femininity α = .80).

### Statistical Analyses

Zero-order and partial correlations were examined to a) rule out confounding effects of BMI, age, and the BSRI scales, and b) test *H_1_*. To analyze moderation effects, a chi-square test was first computed to ascertain that the moderator (gender schematicity) was uncorrelated with the independent variable (sex). Subsequently, MANOVA was used to test *H_2a_* and *H_2b_*, specifically whether gender schematicity moderates sex differences in BII and body satisfaction. The univariate component for BII was followed up with planned sex comparisons at both levels of gender schematicity. The multivariate component of the analysis was more ancillary; its purpose was to explore whether the hypothesized interaction may be mediated by other variables. An additional ANOVA was conducted to rule out specifically perfectionism as a mediator of the hypothesized interaction.

## RESULTS

### Preliminary Analyses and Correlations

Table 1 shows the descriptive statistics for each sex and Pearson product-moment correlations between the study variables. The mean BMI was in the normal range for both female and male participants. As there were no significant sex differences in age, *t*(137) = −1.14, *p* = .25, *d* = 0.02, or BMI, *t*(139) = .14, *p* = .89, *d* = −0.20, the main analyses were collapsed across all participants. Further, participants’ BII scores were not significantly related to the demographic variables of age and BMI. BMI was associated with body satisfaction only.

**TABLE 1:** Means, Standard Deviations, and Intercorrelations for Study Variables

	Male						
Variable	*M*	*SD*	1	2	3	4	5
1. BII	3.03	0.58	—				
2. Perfectionism	1.69	0.82	.41***	—			
3. Body satisfaction	3.67	0.61	−.25**	−.32***	—		
4. BMI	22.73	3.06	.03	−.02	−.34***	—	
5. Age	20.23	2.80	−.04	−.22**	.15	−.08	—
Female *M* (*SD)*			3.25	1.62	3.37	23.65	20.16
			(0.70)	(0.75)	(0.63)	(4.01)	(2.46)

*Note.* Correlation coefficients between variables 1–3 are one-tailed. BII = body image importance.

**p < .01. ***p < .001.

In terms of associations among the main study variables, there were a significant, positive correlation between BII and perfectionism and significant, negative correlations of body satisfaction with BII and perfectionism. However, a one-tailed partial correlation between BII and body satisfaction, controlling for perfectionism, did not reach significance, *r*(138) = −.14, *p* > .05. Partial correlations (two-tailed) between BII and the two continuous BSRI dimensions, controlling for sex, were not significant for either masculinity, *r*(138) = .15, *p* = .07, or femininity, *r*(138) = .16, *p* = .07.

### Moderation Analyses

Table 2 displays the descriptive statistics for participants’ BII and body dissatisfaction scores as a function of sex and gender schematicity. A chi-square test of sex and gender schematicity did not reach significance, χ^2^(1, *N* = 141) = .10, *p* = .75. Thus, a 2 (sex: male, female) × 2 (gender schematicity: gender-schematic, gender-aschematic) between-subjects MANOVA was conducted on BII and body dissatisfaction.

**TABLE 2:** Estimated Marginal Means and Standard Errors for Body Image Measures as a Function of Sex and Gender Schematicity

	Body image importance		Body satisfaction	
Group	*M*	*SE*	*M*	*SE*
Women				
Gender-schematic (*n* = 81)	3.17	.07	3.33	.07
Gender-aschematic (*n* = 30)	3.49	.12	3.50	.11
Men				
Gender-schematic (*n* = 21)	3.13	.14	3.73	.14
Gender-aschematic (*n* = 9)	2.78	.22	3.53	.21

Table 3 shows the univariate and multivariate effects of sex and gender schematicity on the two body image measures. There was a significant main effect of sex on BII that was qualified by a significant Sex × Gender Schematicity interaction. Pairwise comparisons adjusted for multiple comparisons (using the Sidak method) showed that gender-aschematic female participants had significantly higher BII scores than gender-aschematic male participants (*p* < .01; 95% CI [0.21, 1.20]), whereas the BII scores of gender-schematic male and female participants were almost identical (*p* = .84; 95% CI [–0.35, 0.29]). Additional post-hoc comparisons were not examined, due to potential power problems associated with the unbalanced design. Overall, gender-schematicity moderated sex differences in BII. Although sex and gender schematicity had no significant main or interaction effects on body satisfaction, a visual inspection of the data suggests an interaction that may be suppressed by a lack of power.

**TABLE 3:** Multivariate and Univariate Analyses of Variance for Body Image Measures

				Univariate					
	Multivariate			BII			Body satisfaction		
Source	*F*^a^	*p*	η^2^_*p*_	*F*^b^	*p*	η^2^_*p*_	*F*^b^	*p*	η^2^_*p*_
Sex	3.47	.03	.05	6.10	.01	.04	2.44	.12	.02
Gender schematicity	0.02	.99	.00	0.01	.92	.00	0.01	.91	.00
Sex × Gender Schematicity	4.43	.01	.06	5.04	.03	.04	1.70	.19	.01

*Note.* Multivariate *F* ratios were generated from Pillai's statistic. BII = body image importance.

a Multivariate *df* = 2, 136. ^b^Univariate *df* = 1, 137.

There was a significant main effect of sex on the linear combination of BII and body satisfaction that was again qualified by a significant Sex × Gender Schematicity interaction. Box's test did not reach significance, *M* = 10.23, *F*(9, 6692) = 1.08, *p* = .38, indicating equality of covariance matrices. Perfectionism was not entered as a covariate in the analysis, as the homogeneity of regression assumption was violated. Instead, an additional ANOVA of the same independent variables was conducted on perfectionism, but revealed no main or interaction effects. Thus, perfectionism unlikely explains the significant univariate and multivariate effects observed in the main analysis.

## DISCUSSION

The goal of this study was to advance the understanding of individual differences in, and thus elevated levels of BII. The results support the hypothesis that perfectionism would account for BII's relationship with body satisfaction. The moderate correlation between BII and perfectionism is consistent with the transdiagnostic theory of eating disorders (Fairburn et al., 2003), or the co-occurrence of clinical perfectionism and weight and shape overvaluation (e.g., Wade & Bulik, 2007; Wade & Lowes, 2002). Building on findings from clinical samples, this result indicates that perfectionism also explains individual differences in BII within the general population. Thus, even though the phenotypic expressions associated with various levels of BII were not examined, perfectionism may influence the magnitude to which people engage in body change behaviors (i.e., via BII). Also consistent with previous findings is the correlation between BII and body satisfaction (e.g., McCabe & Ricciardelli, 2003; Muris et al., 2005). However, the literature has not taken up a stance on the nature of this relationship. Although perfectionism was previously identified as a correlate of body dissatisfaction as well (Welch et al., 2009), it has not been considered as a common factor of different body image constructs. The present results yield preliminary evidence that BII and body satisfaction are associated as a function of perfectionism.

The hypothesized interaction of sex and gender-schematicity on BII (*H_2a_*) was also observed. Due to an unbalanced design, however, this finding needs to be interpreted with caution. Gender-schematic males and females, who did not differ in BII, may attribute comparable meaning of the body to their own sex. That is, the role that the body plays in self-evaluation may be quite similar for these two groups. Further, being gender-aschematic seems to protect men against elevated BII, which is consistent with research documenting the benefits of belonging to this group (e.g., Bem & Lenney, 1976). A somewhat unexpected result is therefore that gender-aschematic female participants had the highest BII scores. Perfectionism does not appear to mediate the observed interaction, and no moderating effects of gender schematicity on body satisfaction were observed, consistent with *H_2b_*. Yet, the significant multivariate effects indicate that other factors may be implicated in the interaction. A candidate mediator is perceived sociocultural pressure, which is associated with both BII and body satisfaction (e.g., McCabe & Ricciardelli, 2003).

### Limitations and Future Directions

Even though the unbalanced design was partially compensated by using pairwise comparisons adjusted for unequal *ns*, power problems made it unreasonable to examine post-hoc comparisons for the other pairs (e.g., comparing gender-schematicity groups within each sex). Another limitation is the use of a self-report measure to assess gender-schematic processing. Although evidence suggests that the BSRI does a decent job of classifying individuals based on gender schematicity (Bem, 1981b; Schmitt & Millard, 1988), it identifies people who are likely to engage in gender-schematic processing but does not directly measure this construct (Bem, 1981b). A more direct measure that could lead to larger effect sizes and finer distinctions (rather broadly dividing into two groups) is needed. Further research of gender schematicity's role in body image constructs and eating behaviors, using larger samples, seems warranted. BMI was not a central study variable, but the reliance on self-reported weight has limitations. Participants in the overweight and obese groups may have underreported their weight, as the sample proportions in these categories were below national proportions.

The present study was narrower in its focus on cognitive factors implicated in BII. However, researchers have noted the longitudinal stability of shape and weight overvaluation (Cooper & Fairburn, 1993), indicating that people differ consistently in the importance they attach to their bodies. Presumably, the construct extends into the realm of personality and, thus, it may be enlightening to examine the strength and pattern of associations between BII and higher-order personality factors. In this respect, trait perfectionism may be more predictive of consistent individual differences in BII; the more situation-specific cognitive dimension may influence variations within individuals. Research into the relative proportions of BII variance explained by personality and cognitive factors will advance the understanding of the construct. Future research also needs to compare the extent to which different BII constructs mediate the associations between more general cognitive and personality factors and body change behaviors.

### Conclusions

The cognitive factors of perfectionism and gender schematicity appear to be implicated in more specific body image constructs that are pertinent to both non-clinical and clinical populations. Although BII has been fairly resistant to therapy (Cooper & Fairburn, 1993), no adequate modification techniques may have been developed and utilized, as the construct is not particularly well understood. With the aim of alleviating elevated BII, intervention studies may investigate the utility of targeting the superordinate factors of perfectionism and gender schematicity in therapy. The results also shed light on the inconsistent findings concerning sex differences in BII (e.g., Davison & McCabe, 2006; McCabe & Ricciardelli, 2001, 2009; McCabe, Ricciardelli, & Holt, 2010). The presence of sex differences may depend on the relative sample proportions of gender-schematic and gender-aschematic participants. Men's and women's BII scores may be particularly discrepant in populations with high proportions of gender-aschematic individuals.

## Footnotes

1. We thank an anonymous reviewer for stressing that it remains a subject of debate to what extent different dimensions of perfectionism are implicated in eating behaviors and associated body image factors. The different dimensions reflect different levels of measurement (e.g., trait perfectionism refers to behaviors, whereas cognitive perfectionism reflects underlying perfectionstic thoughts). The rationale behind our focus on cognitive perfectionism is that this dimension appeared to be most coherent with the nature of the body image constructs examined in this study.

2. The upper age limit was determined statistically, by excluding those participants whose standardized age exceeded 35 standard deviations.

3. A participant's *t* ratio equals his or her Femininity minus Masculinity scores, multiplied by a conversion factor of 2.322. Bem (1974) derived this constant from two normative samples of 917 students at two different American colleges.
